# Whole Exome Sequencing Reveals a *XPNPEP3* Novel Mutation Causing Nephronophthisis in a Pediatric Patient

**DOI:** 10.29252/ibj.24.6.400

**Published:** 2020-05-31

**Authors:** Rasoul Alizadeh, Sanaz Jamshidi, Mohammad Keramatipour, Parisa Moeinian, Rozita Hosseini, Hasan Otukesh, Saeed Talebi

**Affiliations:** 1 Department of Medical Genetics and Molecular Biology, School of Medicine, Iran University of Medical Sciences, Tehran, Iran;; 2 Department of Medical Genetics, Schools of Medicine, Tehran University of Medical Sciences, Tehran, Iran;; 3 Department of Pediatrics, School of Medicine, Iran University of Medical Sciences, Tehran, Iran;; 4 Ali Asghar Children's Hospital, Tehran, Iran

**Keywords:** Nephronophthisis, Whole exome sequencing, *XPNPEP3*

## Abstract

**Background:**

NPHP is a progressive tubulointestinal kidney condition that demonstrates an AR inheritance pattern. Up to now, more than 20 various genes have been detected for NPHP, with NPHP1 as the first one detected. XPNPEP3 mutation is related to NPHP-like 1 nephropathy and late onset NPHP.

**Methods:**

The proband (index patient) had polyuria, polydipsia and chronic kidney disease and was clinically suspected of NPHP. After the collection of blood sample from proband and her parents, WES was performed to identify the possible variants in the proband from a consanguineous marriage. The functional importance of variants was estimated by bioinformatic analysis. In the affected proband and her parents, Sanger sequencing was conducted for variants’ confirmation and segregation analysis.

**Results:**

Clinical and paraclinical investigations of the patient was not informative. Using WES, we could detect a novel homozygous frameshift mutation in *XPNPEP3* (NM_022098.2: c.719_720insA; p. Q241Tfs*13), and by Sanger sequencing, we demonstrated an insertion in *XPNPEP3*.

**Conclusion:**

The homozygous genotype of the novel p.Q241Tfs*31 variant in *XPNPEP3 *may cause NPHP in the early childhood age.

## INTRODUCTION

Nephronophthisis was first described by Smith and Graham in the mid-40s. This disorder was first called MCKD, but in the early 50's, the term NPHP was first used by Fanconi^[^^1^^]^. NPHP and related diseases are considered as ciliopathy^[^^2^^]^ and lead to ESRD in individuals up to 10 years of age^[^^3^^]^. NPHP is an AR disorder, considered as the most prevalent cause of renal diseases in the first two decades of life, accounting for about 2.4-15% of ESRD^[^^4^^]^. Unlike MCKD, ESRD occurs in fourth decade of life or later^[^^5^^]^. MCKD shows an autosomal dominant pattern, and in this sense, it is distinct from NPHP^[^^2^^]^. 

After the introduction of NPHP by Fanconi, in 1982, around 300 cases were reported, and 10%-15% of them showed extra renal symptoms such as retinitis pigmentosa (Senior-Loken syndrome), cerebellar vermis aplasia (Joubert syndrome), liver fibrosis, skeletal changes, congenital cardiac malformations, obesity, central nervous system abnormalities, and situs inversus^[^^2^^,^^5^^-^^7^^]^. In 1997, *NPHP1* was identified as the first gene in which pathogenic variants can cause NPHP. *NPHP1* mutation, the main cause of NPHP, was detected in almost 20% of the cases. Up to now, pathogenic variants in more than 20 various genes, including NPHP1*, *TMEM216*,* CC2D2A*,* TTC21B*,* TMEM67*,* INPP5E*,* MKS1*,* ARL13B*,* CEP290*, *GLIS2*,* IQCB1*,* NPHP3*,* NPHP4*,* CEP290*,* INVS*, *RPGRIP1L**, **NEK8*,* AHI1, and XPNPEP3^[^^3^^,^^8^^]^*, a*re associated with NPHP^[^^2^^,^^4^^,^^5^^]^*.*

Previous studies have demonstrated that the genes associated with NPHP encode nephrocystin, and the majority of them are mostly located in primary cilia and cilia extended to the kidney tubular lumen; therefore, NPHP was classified as ciliopathies^[^^9^^]^. Based on the incidence of ESRD, the NPHP is categorized into three clinical forms: infantile, juvenile, and adolescent, of which the juvenile form is most common. The juvenile NPHP incidence occurs mostly at age 13, but the symptoms of the disease begin between the ages of four and six^[^^4^^,^^5^^]^. 

Herein, we report the causative variant in the gene belonging to the family of *XPNPEP3*. The encoded protein is localized in the mitochondria of renal cells^[^^10^^]^. Disorders related to *XPNPEP3 *comprise NPHP-like 1 nephropathy and late onset NPHP. Owing to the genetic heterogeneity and various genes involved in NPHP, this disease is a major clinical finding in multiple syndromes such as Senior L*ø*ken, Meckel-Gruber, Cogan, Sensenbrenner, and Joubert^[^^4^^]^. The symptoms of NPHP includes polydipsia, polyuria, growth retardation, anemia, and secondary enuresis^[^^4^^]^. The present study showed a novel homozygous frameshift mutation in *XPNPEP3 *(NM_022098.2: c.719_720insA; Q241Tfs *13) in an Iranian patient using WES. 

## MATERIALS AND METHODS

**Subjects and clinical assessment**

The patient was referred for the genetic diagnosis of NPHP by a medical geneticist. An informed written consent was obtained from the parents of the proband, prior to blood collection from proband and her parents as reference points. Clinical assessment consisted of standard history, physical examination, renal scintigraphy, abdominal and pelvic sonography, and metabolic profiling. 

**DNA extraction**

Peripheral blood samples were collected from the proband (index patient) and her parents for the extraction of genomic DNA by using the Exgene™ Clinic SV kit (GeneAll Biotechnology Co. Ltd., Seoul, Korea), according to the manufacturer’s manual. The DNA from parents was used for the segregation analysis.

**WES**

To identify the underlying genetic causes of the disorder, WES was performed by Macrogen, Korea. Exons of DNA sample were captured using the in-solution SureSelect Target Enrichment System (Agilent, Human All Exon Kits v6; Agilent Technologies, Inc., Santa Clara, CA, USA), followed by a paired-end high-throughput sequencing on reads of 100 bp using Illumina HiSeq 4000 (Illumine Inc., San Diego, CA, USA). A 7.15-gigabase sequence was generated with at least 98.08% coverage for 4×, 91.86% for 20×, and 85.66% for 30× of the sample. The coverage of the target region was 98.93%, and the mean depth was 90.58×. To confirm the candidate variants found in WES, Sanger sequencing was performed.

***In silico*** **pathogenicity assessment of variants**

The obtained variants were annotated with wANNOVAR (http://wannovar.wglab.org/), and the process was followed by the variant frequency analysis (i.e. minor allele frequency <0.02) reported in the single nucleotide polymorphism database (http://www.ncbi.nlm.nih.gov/SNP/), the 1000 genome project dataset (http://www. 1000genomes.org), the ESP6500, gnomAD, and ExAC (http://exac.broadinstitute.org). Bioinformatics analyses were conducted to confirm the pathogenic novel variants obtained from WES. The damaging effects of the novel variant were assessed by using bioinformatics tools, including SIFT (sift.jcvi.org)^[^^11^^]^, PolyPhen-2 (http://genetics.bwh.harvard.edu/pph2)^[^^12^^]^, CADD^[^^13^^]^, and Mutation Taster (http://www. mutationtaster.org)^[^^14^^]^, based on Standards and Guidelines for the Interpretation of Sequencing Variants (ACMG-AMP guidelines)^[^^15^^]^. Among the detected variants, frameshift variants or variants predicted to be damaging were considered as the most promising candidates. PubMed and OMIM databases were reviewed for publications regarding candidate genes as well as functional and expression data. Sequencing the limited coding region of *XPNPEP3 *(sequence reference: NM_022098.2) as well as Sanger sequencing confirmed our result and demonstrated an insertion in *XPNPEP3 *in the affected proband.

**Ethical statement**

The above-mentioned sampling or treatment or both protocols were approved by the Research Ethics Committee of Iran University of Medical of Medical Sciences, Tehran, Iran (Ethical code: IR.IUMS.FMD. REC.1399.144). An informed consent form was obtained from the parents of the proband.

## RESULTS

**Clinical findings**

The proband case was a 13-year-old female who was born via normal vaginal delivery from an 18-year-old mother. The parents were first-cousin. In prenatal history, threats of abortion occurred at 6^th^ months of pregnancy, and there was oligohydramnios at the third trimester of gestation. By 6^th^ month of age, although she had normal neurodevelopment, growth failure and anemia were obvious. The proband showed polyurea and polydipsia as well as chronic kidney disease at 3^rd^ years of age. Renal ultrasonography indicated an increased parenchymal echogenicity, and the size of kidneys was within the lower limit of normal. 

**Genetic findings**

**Fig. 1:**
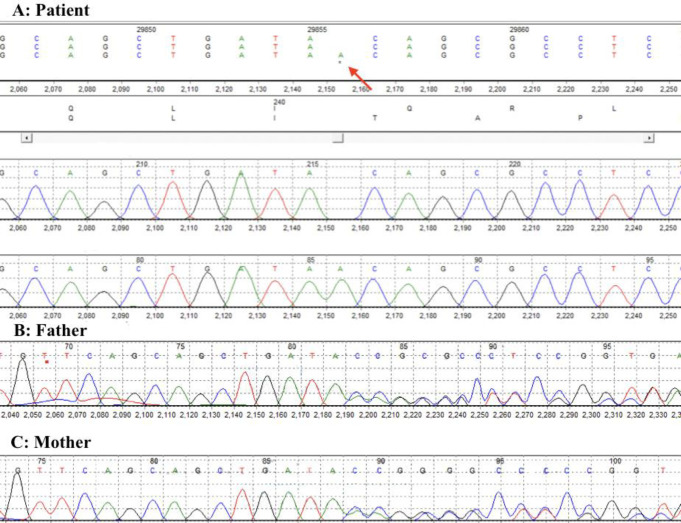
*Sequencing chromatograms of the proband and her parents* *showing* *an insertion of A in *XPNPEP3

WES was performed on proband by focusing on the genes involved in ciliopathies, especially NPHP. According to the data obtained from WES, 11644 indel variations and 104245 single nucleotide variants were detected. We applied filtration process on exome data sequence to define the pathogenic mutation. Finally, by using the zygosity and CADD phred score (cut-off = 15), we arranged the filtered variants. We detected 18 homozygous variants in genes such as *XPNPEP3*, *ACO2*, *DNAH2, MUC4, DARC, SLC25A5, KCNM1*, *ARAF*, and *MPST*. *XPNPEP3 *that caused NPHP was the only mutation matched with the clinical findings of the proband. The mutation of *XPNPEP3* gene was a frameshift in exon 4 (NM_022098.2: c.719_720insA; Q241Tfs*13). Also, Mutation Taster predicted that the mutated *XPNPEP3* was a disease-causing variant. The identified variant was absent in the 1 k Human Genome Database, the ESP6500, gnomAD, and ExAC.  Findings from WES were confirmed by Sanger sequencing (Fig. 1).

## DISCUSSION

NPHP is an AR cystic kidney disease, leading to renal fibrosis and ESRD in affected cases. In addition to symptoms of kidney, nearly 15% of NPHP cases manifest some external symptoms of ciliopathy, including retinal defects, liver fibrosis, skeletal abnormalities, and brain developmental disorders^[^^1^^,^^9^^]^. The diagnosis of NPHP requires clinical characteristics (a renal concentrating defect that leads to polyuria and polydipsia) that have to be confirmed by the biochemical assessment (urine test), renal ultrasound scan, detection of mutation in NPHP- related genes by NGS and WES, and renal biopsy (when diagnosis is not clear or genetic diagnosis is impossible)^[^^4^^,^^16^^]^.

In the present study, WES was used to investigate the genetic cause of a case suspected of NPHP, in which chronic kidney disease and polyurea and polydipsia were not adequately specific for the NPHP diagnosis. We conducted WES on the affected proband, in reference to her parents,to find potentially damaging gene variants and also homozygous variants. Analysis of WES revealed unknown novel homozygous frameshift mutation (NM_022098.2: c.719_720insA; p.Q241Tfs*13) in exon 4 of the *XPNPEP3 *gene associated with NPHP. This frameshift mutation was shown to disrupt the normal reading frame, leading to a PTC. PTC is recognized by the nonsense-mediated mRNA decay, a mechanism that degrades mRNA harboring PTC^[^^17^^]^. 

Based on our bioinformatics survey, p.Q241Tfs*13 was the probable pathogenic candidate variant that was identified by WES. This variant did not exist in 1000 genome project databases and ExAC. The allele also was not found in gnomAD browser with any reported homozygotes. Referring to the latest ACMG-AMP guidelines scoring system/classification, this variant includes one very strong pathogenic evidence (PVS1 score) for null variant (frameshift, affecting gene *XPNPEP3 *which is a known inducer of disease), one moderate pathogenic (PM2 score), and one supporting pathogenic (PP4 score) evidence. Therefore, according to the ACMG-AMP guidelines, this variant may be classified as a pathogenic variant.

In conclusion, our findings revealed that the p.Q241Tfs*13 in *XPNPEP3* is possibly one of the potential pathogenic variants for NPHP.

## CONFLICT OF INTEREST

None declared.
